# Continuous 14 Day Infusional Ifosfamide for Management of Soft-Tissue and Bone Sarcoma: A Single Centre Retrospective Cohort Analysis

**DOI:** 10.3390/cancers12113408

**Published:** 2020-11-17

**Authors:** Thomas J. Carter, Marina Milic, Joanna McDerra, Anne McTiernan, Mahbubl Ahmed, Vasilios Karavasilis, Maria Michelagnoli, Rachael Windsor, Beatrice Seddon, Jeremy Whelan, Palma Dileo, Sandra J. Strauss

**Affiliations:** 1 London Sarcoma Service, University College London Hospital, Euston Road, London NW1 2PG, UK; thomascarter@nhs.net (T.J.C.); marinamilic@nhs.net (M.M.); j.mcderra@nhs.net (J.M.); anne.mctiernan@nhs.net (A.M.); mahbubl.ahmed@nhs.net (M.A.); vasilios.karavasilis@nhs.net (V.K.); maria.michelagnoli@nhs.net (M.M.); rachael.windsor@nhs.net (R.W.); beatrice.seddon@nhs.net (B.S.); Jeremy.whelan@nhs.net (J.W.); palma.dileo@nhs.net (P.D.); 2 Research Department of Oncology, UCL Cancer Institute, 70 Huntley Street, London WC1E 6BT, UK

**Keywords:** infusional ifosfamide, bone sarcoma, soft-tissue sarcoma, chemotherapy

## Abstract

**Simple Summary:**

Ifosfamide is commonly used to treat patients with soft-tissue and bone sarcoma, with greater efficacy observed with higher doses that generally require inpatient treatment and may result in significant myelosuppression and renal toxicity. In the palliative setting, continuous infusional ifosfamide (14 g/m^2^/14 days) is increasingly employed in an attempt to mitigate toxicity, and for ease of administration as an outpatient regimen. This study describes the efficacy and toxicity profile of 14-day continuous infusional ifosfamide in adult and teenage young adult (TYA) patients with relapsed or metastatic soft-tissue and bone sarcoma.

**Abstract:**

Ifosfamide is used to treat soft-tissue sarcoma (STS) and bone sarcoma (BS), with improved efficacy at doses above 9 g/m^2^/cycle. To mitigate treatment-associated toxicity with higher doses, continuous infusional ifosfamide is increasingly used. However, clinical outcome data remain limited. Single-centre retrospective analysis of patients treated with four-weekly infusional ifosfamide (14 g/m^2^/14d) between August 2012 and February 2019 was conducted. Radiological response, progression-free survival (PFS), overall survival (OS) and toxicity were evaluated. Eighty patients were treated—46 with STS and 34 with BS. Patients received a median of three cycles of infusional ifosfamide (1–24). Overall disease control rate (DCR) in STS was 50% (23 of 46 patients), with a median PFS of 3.8 months, and median OS of 13.0 months. In synovial sarcoma (SS), DCR was 80% (12/15), median PFS 8.1 months and median OS 20.9 months. Overall DCR in BS (34 patients) was 30%, with a median PFS of 2.5 months and median OS of 6.2 months. Five patients (6%) stopped treatment due to toxicity alone within the first two cycles. A further 10 patients stopped treatment due to toxicity during later treatment cycles (12%) and 18 patients (23%) required dose modification. Forty-five patients (56%) experienced grade (G) 3/4 haematological toxicity, with 12 episodes of febrile neutropenia and one treatment-related death. Twenty-seven patients (34%) experienced G3/4 non-haematological toxicity, most commonly nausea and vomiting (10, 13%). In summary, infusional ifosfamide has efficacy in STS, most notable in SS. Benefit appears limited in BS. Treatment is associated with toxicity that requires specialist supportive care.

## 1. Introduction

Ifosfamide is a member of the oxazaphosphorine family of alkylating agents and is an established systemic treatment in both soft-tissue sarcomas (STS) and bone sarcomas (BS), rare heterogenous malignant tumours of mesenchymal origin [1]. In STS, ifosfamide is often given in combination with doxorubicin as first-line treatment for locally advanced disease [2]. Whilst the combination does not confer an overall survival advantage over doxorubicin alone, the addition of ifosfamide significantly increases PFS and doubles radiological responses [3]. In BS, ifosfamide is a component of first-line chemotherapy in Ewing sarcoma (ES) [4], whilst in osteosarcoma, ifosfamide is commonly used in combination with etoposide in patients at first relapse [5].

For STS or BS patients with further relapse or metastatic disease not amenable to surgical management, there are few effective systemic treatment options and median overall survival (OS) remains poor. High-dose ifosfamide (>9 g/m^2^/cycle) is an active agent but its use can be challenging, particularly in the palliative setting, because of toxicities including myelosupression, nephrotoxicity and neurotoxicity. One attempt to mitigate these toxicities is through the use of continuous infusional ifosfamide [6,7,8], which can be administered *via* a continuous ambulatory delivery device (CADD) [9], concurrently with mesna to minimise the risk of urothelial toxicity [10,11]. In addition to better tolerability, it has been proposed that this delivery method may possess superior cytotoxicity [12] and can be used as an outpatient regimen, whereas conventional fractionated ifosfamide is predominantly used in the ambulatory setting.

In recent years, continuous infusional ifosfamide has become more common as a treatment for patients with relapsed or metastatic STS and BS, although outcome data to justify the greater use of this schedule, is limited. The aim of this study was to retrospectively assess the efficacy, outcome and toxicity for patients with both BS and STS treated with infusional ifosfamide (14 g/m^2^/cycle over 14 day administered via CADD) at a large reference centre.

## 2. Results

### 2.1. Patient Characteristics

**Figure 1:**
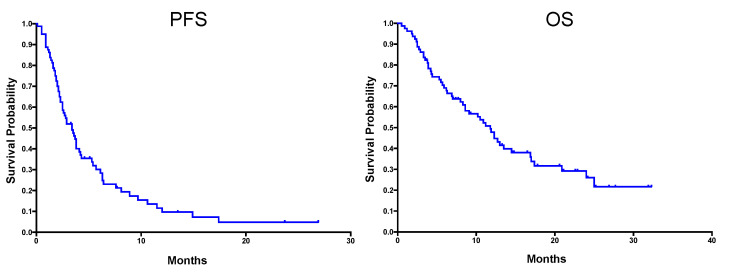
Kaplan–Meier (KM) curves showing PFS (left) and OS (right) for all 80 patients—46 patients with STS and 34 with BS. Median PFS was 3.4 months and median OS was 11.8 months.

A total of 80 patients were identified, of whom 46 had a diagnosis of relapsed or metastatic STS, and 34 had a diagnosis of relapsed or metastatic BS (Table 1). For both the STS and BS cohorts, patients had a median of one site of metastatic disease, with lungs as the most common site of metastatic spread. In the STS cohort, the median age was 43 years, and patients had received a median of one prior line of systemic treatment (range 0–4). The most common STS histological subtypes were synovial sarcoma (SS) (15 patients) and liposarcoma (LPS) (13 patients); 24 (52%) had received prior ifosfamide. In BS, median age was 23 years, with patients receiving a median of 2 (range 0–5) previous lines of systemic treatment, with 23 (68%) patients having received prior ifosfamide treatment. Ewing sarcoma (ES) was the most common histological subtype (16 patients). Patients with STS received a median of four cycles of infusional ifosfamide (range 1–24) and BS patients received a median of three cycles (range 1–8). For the whole cohort, the DCR was 41% (33 patients), with a median PFS (mPFS) of 3.4 months (m), and a median OS (mOS) of 11.8 m (Figure 1).

### 2.2. Soft-Tissue Sarcoma

**Figure 2:**
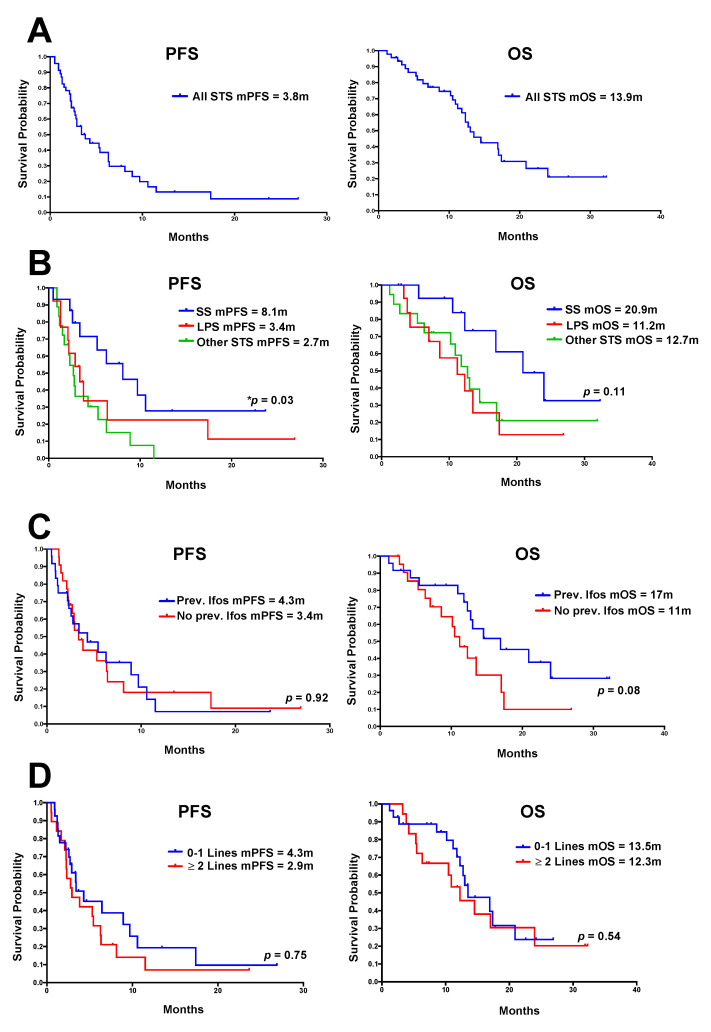
Kaplan–Meier (KM) curves showing PFS and OS in patients with STS. (**A**) All STS patients; (**B**) compared by histological subtype; (**C**) compared by prior ifosfamide exposure; (**D**) compared based on the number of previous chemotherapy lines. Median progression-free survival (mPFS) and overall survival (mOS) for each subgroup are shown. SS = synovial sarcoma, LPS = liposarcoma. Statistical analysis was performed using log-rank (Mantel–Cox) test to compare survival curves. * Statistically significant difference between KM curves.

For all STS patients, DCR was 50% (23 of 46 patients), including 13 with an imaging response and 10 patients with stable disease as their best response. Eleven patients had radiologically confirmed disease progression, and of the remaining 12 patients who were not evaluated, four showed evidence of clinical disease progression alone, three stopped treatment due to toxicity alone, and five had toxicity together with evidence of clinical progression. Six patients went on to have consolidation treatment, with details from these patients provided later. For all STS patients, mPFS was 3.8 m, and mOS was 13.9 m (Figure 2A).

When analysed by histological subtype, a DCR of 80% was observed for SS patients, with mPFS of 8.1 m (*p* = 0.03), and mOS of 20.9 m (Figure 2B). DCR was 38% (5/13) for LPS patients, including 3/7 patients with de-differentiated LPS (DDLPS) and 1/1 patient with pleomorphic LPS and 1/2 patients with well-differentiated LPS (WDLPS). For the LPS patients, mPFS was 3.4 m and mOS was 11.2 m. Prior ifosfamide exposure did not appear to impact on likelihood of patient benefit (Figure 2C). No difference was observed between the KM curves for patients who were treated with 0–1 previous lines of chemotherapy, compared with those treated with >2 previous lines (Figure 2D).

**Figure 3:**
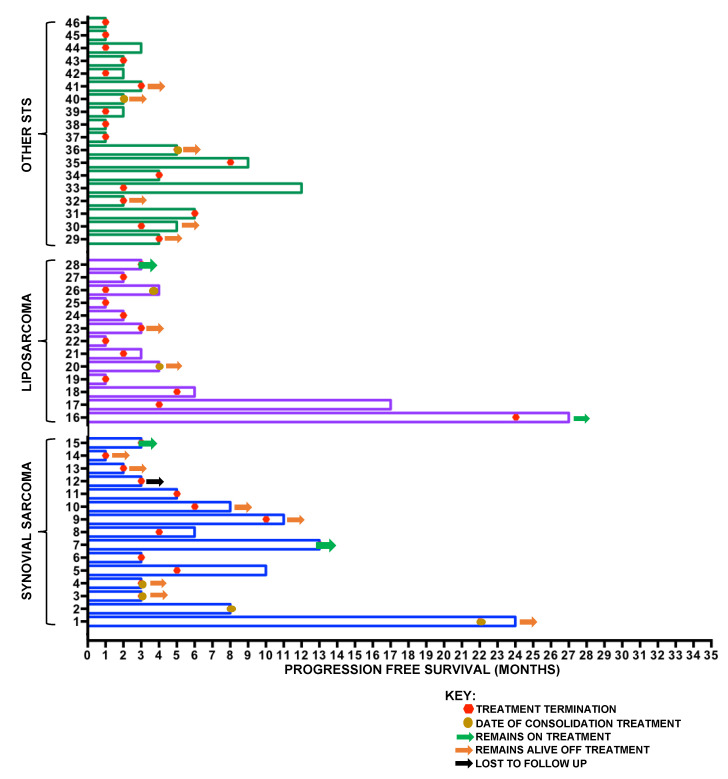
Swimmer plot of STS cohort demonstrating PFS, timing of treatment cessation, and patient status at the time of analysis. Patients 1–15 represent those with synovial sarcoma, patients 16–28 represent those with liposarcoma, whilst patients 19–46 represent those with other STS subtypes.

To represent individual patient results within the STS cohort, data were subsequently analysed using a swimmer plot (Figure 3) to demonstrate for each patient the PFS and timing of treatment cessation as well as highlighting those who remained on treatment or received consolidation treatment following infusional ifosfamide.

### 2.3. Bone Sarcoma

**Figure 4:**
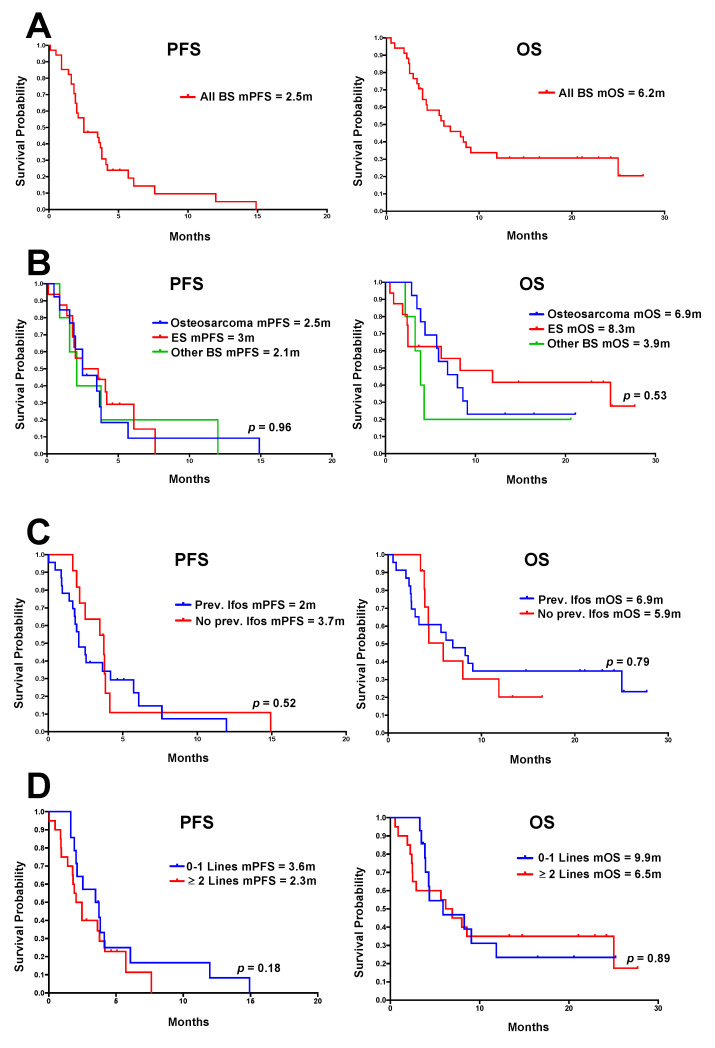
Kaplan–Meier (KM) curves showing PFS and OS in patients with BS. (**A**) All BS patients; (**B**) compared by histological subtype; (**C**) compared by prior ifosfamide exposure; (**D**) compared based on the number of previous chemotherapy lines. Median progression-free survival (mPFS) and overall survival (mOS) for each subgroup are shown. ES = Ewing sarcoma. Statistical analysis was performed using log-rank (Mantel–Cox) test to compare survival curves.

In the cohort of 34 BS patients, overall DCR was 30% (10 patients), six patients with imaging response, and four with disease stability as best response. Of the remaining patients, 50% (17) had confirmed radiological disease progression, and of seven non-assessed patients, five had evidence of clinical progression, one patient experienced toxicity in combination with clinical progression, and two stopped treatment due to toxicity alone, including one treatment-related death. Three patients had consolidation treatment, with details given later. Median PFS was 2.5 m and mOS was 6.2 m (Figure 4A).

**Figure 5:**
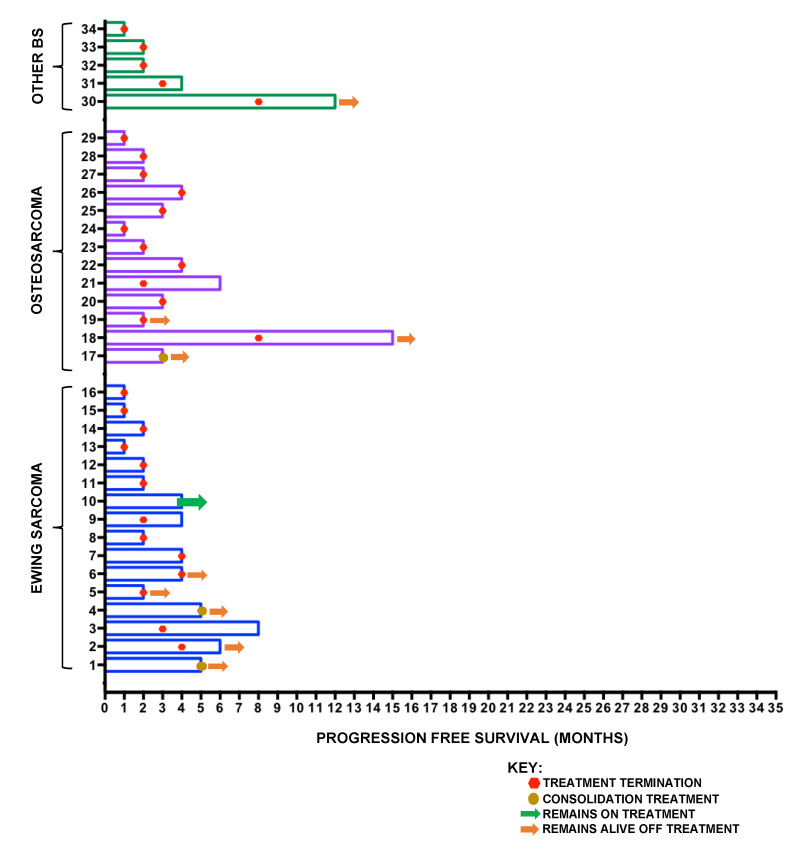
Swimmer plot of BS cohort demonstrating PFS and timing of treatment termination, along with details of their current status at the time of analysis.

For patients with ES (16 patients), DCR was 38% (six patients), mPFS was 3 m and mOS was 8.3 m (Figure 4B). For patients with osteosarcoma (13 patients), DCR was 15% (two patients), mPFS 2.5 m and mOS 6.9 m. For the remaining patients (five), DCR was 40% (two patients), mPFS was 2.1 m and mOS was 3.9 m, including one patient with a diagnosis of mesenchymal chondrosarcoma who progressed 12 months following treatment initiation. No significant differences between PFS or OS were observed in the histological subtype analysis. Previous exposure to ifosfamide (Figure 4C) or number of lines of prior chemotherapy (Figure 4D) did not impact on benefit. A swimmer plot showing PFS, timing of treatment cessation and current status for individual patients is shown in Figure 5.

### 2.4. Consolidation Treatment after Infusional Ifosfamide

Nine patients received consolidation treatment (radiotherapy and/or surgery) following infusional ifosfamide (Table 2). Patients had either oligometastatic disease (four patients) or local recurrence (five patients) with SS (four patients) and ES (two patients) the most common histological subtypes treated. The median number of cycles of infusional ifosfamide given was five (range 3–22), and all patients achieved disease control either with radiological and/or metabolic improvement (67%) or stable disease (33%). Median OS for these patients at the date of analysis was 24 m (range 12–32).

### 2.5. Toxicity Analysis

Toxicity data are summarised in Table 3. Thirty-eight patients (48%) attended an emergency department or were admitted to hospital whilst on treatment, including two intensive care admissions, one for neutropenic sepsis and one for suspected ifosfamide encephalopathy with concurrent severe electrolyte disturbance and acute kidney injury (AKI). Eighteen patients (28%) required dose modifications as a result of toxicity including four patients who were switched to standard high-dose fractionated ifosfamide. Eleven patients (14%) stopped treatment due to toxicity within the first two cycles—five due to toxicity alone and six patients with concurrent evidence of clinical disease progression. Of the 11 patients who stopped treatment within the first two cycles, five patients experienced confirmed or suspected encephalopathy (two patients at G2, three patients at G3/4), two experienced G3 nausea and/or vomiting, two experienced G3 fatigue, and two suffered >G3 infections including one episode of G4 neutropenic sepsis. A further 10 patients stopped treatment due to toxicity during later treatment cycles (12%)—six due to haematological toxicity, two due to severe fatigue and two due to persistent nausea/vomiting.

The overall rate of grade 2 or above (G ≥ 2) haematological toxicity was 78%, with 56% of patients experiencing G3/4 toxicity. Neutropenia was the most common haematological toxicity, with 31% of patients experiencing G3/4 neutropenia. Twelve patients (15%) had neutropenic sepsis or febrile neutropenia, including one patient who died of acute respiratory distress syndrome as a complication of protracted neutropenic sepsis. In total, 47 patients (59%) experienced at least one G ≥ 2 non-haematological toxicity the most common being nausea and/or vomiting, with an overall G ≥ 2 rate of 26% (20 patients). The second most common toxicity was fatigue (nine patients, 12%). Rates of suspected or confirmed encephalopathy were 8% (six patients), with the majority of cases (83%, five patients) occurring in the first two cycles. Three patients suffered G3/4 symptoms, including one patient requiring admission to the intensive treatment unit (ITU) for management; only one patient with G2 symptoms was re-challenged with ifosfamide. Three patients (4%) experienced G2 haematuria, and no patients experienced G3/4 urothelial toxicity, although two patients were diagnosed with transient G3/4 AKI, and two patients experienced G3 electrolyte disturbance. Toxicity was similar across TYA and adult populations with 67% of TYA patients experiencing at least one G ≥ 2 non-haematological toxicity (18/27), compared to 55% (29/53) of adult patients.

## 3. Discussion

These data provide a comprehensive analysis of the benefit and tolerability of continuous infusional ifosfamide (14 g/14 days) across patients with BS and STS. For purpose of analysis, results were compared to existing studies utilising the same dosing schedule [13,14], as well as studies utilising alternative ifosfamide dosing schedules, in the relapsed sarcoma setting. Presented results are interpreted cautiously, recognising that treatment was given outside of the clinical trial setting, and therefore RECIST reporting was not available and adverse-event data were retrospectively collated. Previously reported mPFS for mixed BS and STS cohorts treated with infusional ifosfamide have been 2.9 m in a mixed cohort of 30 patients [15] and 3 m in a mixed cohort of 51 patients [16], consistent with the mPFS of 3.4 m in this cohort. Median OS in a patient group treated with ifosfamide at 2 g/m^2^/6 days every 3 weeks was reported as 8.7 m (30 patients) [15], compared to 11.8 m in the present cohort. Due to the well-characterised differences in clinical behaviour between STS and BS, further detailed response and efficacy analysis was performed separately for the STS and BS cohorts, with toxicity data subsequently analysed for the whole cohort.

In a previously published cohort of 35 STS patients treated with 14-day infusional ifosfamide, mPFS was reported as 4.2 m, with mOS of 11.2 m [13], once again consistent with our results. When analysed by histological subtype, our data demonstrate greatest efficacy in patients with SS, with a DCR of 80%, mPFS of 8.1 m and mOS of 20.9 m. Furthermore, prior exposure to ifosfamide did not predict lower response rates in SS, in keeping with a previous study demonstrating that ifosfamide re-challenge was most effective in patients with SS, achieving a disease control rate of 49% (14 out of 29 patients) [17]. These findings confirm that prior ifosfamide exposure should not be a contra-indication to treatment with infusional ifosfamide in STS, especially in patients with relapsed SS.

Infusional ifosfamide has also been demonstrated to be of benefit in patients with advanced WD/DDLPS, with a DCR of 69% in 28 patients, including nine with radiological improvement, and 11 with disease stability as best response [14]. In that study, all patients with radiological improvement had DDLPS, with four WDLPS and nine DDLPS patients achieving disease stability with a PFS of 7.4 m, compared to 3.4 m in our LPS cohort. This suggests that infusional ifosfamide is more active in patients with DDLPS, reflected in the findings presented here with a DCR of 43% (three out of seven) in DDLPS. Differences in mPFS between these cohorts may be explained by the inclusion of three patients with myxoid liposarcoma in our cohort, all of whom progressed on treatment. In addition, in this histology and particularly in WDLPS, radiological stability could represent the natural history of indolent disease [18,19]. Furthermore, there is emerging evidence that assessing response using Choi criteria [20] may be more predictive of benefit than conventional response assessment [21]. Despite these limitations, we believe the length of time patients remained on treatment is reflective of continued clinical benefit as assessed by the clinical team.

Literature on the use of infusional ifosfamide in BS populations is more limited than that of STS, with the majority of reported studies performed on heterogeneous groups containing both BS and STS patients [15,16], or extrapolated from studies of conventional high-dose ifosfamide in paediatric populations [22]. Grouped outcomes in our BS cohort are comparable to published studies with reported mPFS values of 2.9 m and 3 m [15,16], compared to 2.5 m in our cohort. However, it is more clinically relevant to analyse outcomes for osteosarcoma and Ewing sarcoma separately, given the different underlying biology of these two diseases.

For patients with ES (16 patients), six had clinical benefit including five with radiological/metabolic improvement, and one with stable disease. These findings are consistent with previous studies, including one study of 14-day infusional ifosfamide in 14 paediatric patients [23]. Three patients within this study had a diagnosis of ES and all three gained benefit, including two with radiological improvement. In addition, infusional ifosfamide enabled one of these patients to have surgery as consolidation treatment for residual disease. The role of re-challenge with infusional ifosfamide in ES is further supported by published reports on the use of high-dose ifosfamide (15 g/m^2^/5 days) in relapsed or progressive ES, with clinical benefit reported as 54% (15/28) [24] and 62% (23/37), respectively [25]. In these two cohorts, rates of haematological toxicity were higher than those presently reported, with rates of G4 neutropenia 97% in one study, and admission rates for febrile neutropenia of 77% in the other. Furthermore, whilst the final results of the rEEcur study are awaited, high-dose ifosfamide remains one of the open study arms [26].

The efficacy for use of infusional infosfamide in osteosarcoma (13 patients) appears less favourable, with only two patients (15%) gaining clinical benefit—one with improvement and one with stable disease. In a previous study using 14 day infusional ifosfamide, two patients with osteosarcoma were included, both of whom experienced PD on treatment [23], whilst in separate study using an alternative dosing schedule (14 g/m^2^ over 6 days), a complete response was seen in a patient with metastatic osteosarcoma who was refractory to standard-dose ifosfamide [27]. In contrast, studies investigating conventional high-dose ifosfamide (3 g/m^2^/day for 5 days) in relapsed metastatic osteosarcoma have reported more favourable response rates, especially in paediatric patients (43%; 9/21) and in those with lung-only metastatic disease (21%; 9/35) [28]. Within that cohort, a number of patients underwent consolidation surgery following high-dose ifosfamide with favourable 1-year OS rates in patients achieving surgical complete response [28].

Consolidation treatment following infusional ifosfamide has been presented previously in three children (one with ES and two with SS) following radiological responses to treatment with favourable survival outcomes [23]. We present a total of nine patients—six with STS and three with BS, including four patients with SS and two with ES. In this group, mOS following definitive local therapy was found to be 24 m, and while the numbers of patients presented is small, these data support a role for infusional ifosfamide in selected patients with oligometastatic disease or local recurrence, prior to definitive local therapy.

Toxicity within this cohort is consistent with previous studies on the use of infusional ifosfamide. A G2–4 neutropenia rate of 33% is similar to previous studies reporting rates of 43% G3–4 neutropenia (30 patients) and 53% all-grade neutropenia (51 patients) [15,16] These differences do not describe the use of GCSF, the use of which has increased in recent years. A febrile neutropenia rate of 15%, was significantly lower than with high-dose fractionated ifosfamide given in paediatric populations [24,25]. The most commonly reported non-haematological toxicities were nausea and vomiting and fatigue, findings consistent with previously reported cohorts [13]. Moreover, a rate of 8% for suspected or confirmed encephalopathy is comparable to previous reports of incidences of 4%, 7%, 11% and 17% and no sustained renal toxicity was observed [13,14,15,16]. Toxicity rates were similar between STS and BS cohorts, and between younger TYA and adult patients. In our cohort we reported one on-treatment death from ARDS following a prolonged ITU admission with neutropenic sepsis. On-treatment deaths with infusional ifosfamide are rare, but important, with two other cohorts experiencing one on-treatment death each [15,16], consistent with the experience presented here.

## 4. Materials and Methods

A retrospective analysis was performed of 80 STS and BS patients treated with at least one cycle of 4-weekly infusional ifosfamide (14 g/m^2^/cycle; 14 days on treatment, 14 days off treatment) at UCLH between August 2012 and February 2019. Selected patients were treated with palliative intent, in the outpatient setting. Patients received ifosfamide via continuous infusion via continuous ambulatory delivery device with co-administration of mesna (given at 1:1 g:g ratio). Infusers were changed mid-way through the 14-day cycle, with supportive anti-emetics administered orally throughout treatment. In line with current ASCO guidelines, granulocyte-colony stimulating factor (GCSF) was not given at commencement of therapy, but added if patients experienced febrile neutropenia, or neutropenia delaying subsequent treatment [29]. Patients prescribed infusional ifosfamide were identified from the hospital chemotherapy prescribing database, with ifosfamide prescribing data collected from there, and additional data collected from hospital records including clinical letters, digital pathology and radiology results systems. Data collection was in accordance with local ethical standards [30].

Patients underwent standard of care imaging after every two to three cycles of treatment, most commonly in the format of computed tomography, with three patients undergoing response assessment using positron emission tomography/computed tomography (PET/CT) assessment. Radiological assessment was performed using contemporary radiology reports from specialist musculoskeletal radiologists. As patients were not treated within a clinical trial, RECIST 1.1 reporting was not available. Clinical outcomes were assessed using disease control rate (DCR); defined as the proportion of patients with evidence of response or stable disease (or SUVmax improvement on PET/CT), and without disease progression. PFS was calculated from the date of treatment initiation to the date of confirmed clinical or radiological progression or death, and patients who received consolidation treatment were censored at the time of this therapy. OS was calculated from the date of treatment initiation to death from any cause, and patients were censored at last clinical encounter if still alive at the time of analysis. Kaplan–Meier methods were used to calculate median PFS and OS for patient groups, with graphical analysis performed using GraphPad Prism 6 software. Statistical analysis was performed using log-rank (Mantel–Cox) test to compare survival curves. When collating haematological toxicity, each patient was counted once for their most severe grade for each toxicity. Data for G ≥ 2 haematological and non-haematological toxicities were collated from medical notes and graded utilising the CTCAE v4.0 criteria.

## 5. Conclusions

To the best of our knowledge, this cohort represents the largest single cohort of relapsed or metastatic sarcoma patients treated with infusional ifosfamide. This regimen offers an advantage over high-dose fractionated ifosfamide, as treatment can be conveniently given as an outpatient. In addition, our study confirms lower rates of febrile neutropenia, renal and neurological toxicity with infusional ifosfamide when compared with high-dose fractionated treatment. These findings provide a sound rationale for use of infusional ifosfamide in the palliative setting and demonstrate it to be a valuable treatment option in selected patients with relapsed or metastatic sarcoma. Notable clinical activity was observed in SS and WD/DDLPS with subsets of patients benefitting for prolonged periods, including three patients on treatment for over 1 year—two patients with SS and one with WDLPS. In BS, the role of infusional ifosfamide appears more limited, with some support for benefit in Ewing sarcoma, and a role for patients with oligometastatic disease who are eligible for consolidation therapy with a different modality. These findings highlight the importance of multi-disciplinary care for patients with advanced disease, especially in those being considered for consolidation treatments. Reported toxicity appears consistent with previously published patient cohorts. Whilst toxicity is generally predictable, it can be significant, and so should be a factor considered when making treatment decisions for palliative patients.

## Figures and Tables

**Table 1:** Patient demographics, including histology for soft-tissue sarcoma (STS) and bone sarcoma (BS) cohorts.

Demographic	Soft-Tissue Sarcoma (STS)	Bone Sarcoma (BS)
Number of Patients	46	34
Histology	Synovial sarcoma—15 Liposarcoma *—13 Spindle cell sarcoma—4 MPNST—3 Epithelioid sarcoma—3 Other **—8	Ewing Sarcoma—16 Osteosarcoma—13 Mesenchymal chondrosarcoma—3 De-differentiated chondrosarcoma—1 High-grade spindle cell sarcoma, NOS—1
Median Age in Years (range)	43 (18–72)	23 (12–64)
% Male	48%	62%
Number of Metastatic Sites	1 (0–2)	1 (0–2)
Median Previous Chemotherapy Lines (range)	1 (0–4)	2 (0–5)
Prior Ifosfamide	24 (52%)	23 (68%)

* Liposarcoma: seven patients with de-differentiated liposarcoma, three with myxoid liposarcoma, two with well-differentiated liposarcoma and one with pleomorphic liposarcoma. MPNST: malignant peripheral nerve sheath tumour. NOS: not otherwise specified. ** Other STS: myxofibrosarcoma (2) and leiomyosarcoma (2).

**Table 2:** Summary of patients who underwent consolidation treatment following treatment with infusional ifosfamide (Inf-Ifos).

Pt	Diagnosis	Recurrence	Cycles of Inf-Ifos	Radiological Response	Treatment	Current Status *	Overall Survival *
1	ES	Isolated Bone	6	Radiological/Metabolic Improvement	Surgery	AWD	28 m
2	ES	Local	5	Radiological/Metabolic Improvement	RT	AWD	24 m
3	Osteosarcoma	Mediastinal	4	Radiological/Metabolic Improvement	RT + Surgery	NED	21 m
4	SS	Lung	8	Radiological Improvement	RT	AWD/Lost to Follow Up	24 m
5	SS	Lung	22	Radiological Improvement	Surgery	AWD	32 m
6	SS	Local	3	Radiological Improvement	RT + Surgery	Further RT and Now NED	24 m
7	SS	Local	3	Stable Disease	Surgery	NED	23 m
8	De-diff LPS	Local	4	Stable Disease	RT	NED	12 m
9	MPNST	Local	5	Stable Disease	RT + Surgery	NED	13 m

PD = progressive disease, m = months. * At date of analysis. RT = radiotherapy, AWD = alive with (active) disease, and NED = no evidence of active disease.

**Table 3:** Rates of CTCAE v.4.0 G ≥ 2 haematological and non-haematological adverse events in patients treated with at least one cycle of infusional ifosfamide (n = 80).

Adverse Event	Grade 2 N (%)	Grade ¾ N (%)
Haematological		
All	17 (21%)	45 (56%)
Anaemia	13 (16%)	8 (10%)
Thrombocytopenia	2 (2%)	12 (15%)
Neutropenia	2 (2%)	25 (31%)
Non-Haematological		
All	32 (40%)	27 (34%)
Fatigue	7 (9%)	2 (3%)
Nausea/Vomiting	10 (13%)	10 (13%)
Infection	5 (6%)	7 (9%)
Encephalopathy/Confusion	3 (4%)	3 (4%)
Haematuria	3 (4%)	Nil
AKI	Nil	2 (3%)
Electrolyte Disturbance	1 (1%)	2 (3%)
Other *	3 (4%)	1 (1%)

AKI = acute kidney injury. * Other G2 = diarrhoea, oedema, and bone pain. * Other G3/4 = haemorrhage.

## References

[1] Tascilar M., Loos W.J., Seynaeve C., Verweij J., Sleijfer S. (2007). The Pharmacologic Basis of Ifosfamide Use in Adult Patients with Advanced Soft Tissue Sarcomas. Oncologist.

[2] Seddon B. (2016). First-line treatment in advanced or metastatic disease: One size fits all or adapted to specific histiotypes?. Curr. Opin. Oncol..

[3] Judson I., Verweij J., Gelderblom H., Hartmann J.T., Schöffski P., Blay J.-Y., Kerst J.M., Sufliarsky J., Whelan J., Hohenberger P. (2014). Doxorubicin alone versus intensified doxorubicin plus ifosfamide for first-line treatment of advanced or metastatic soft-tissue sarcoma: A randomised controlled phase 3 trial. Lancet Oncol..

[4] Gaspar N., Hawkins D.S., Dirksen U., Lewis I.J., Ferrari S., Le Deley M.-C., Kovar H., Grimer R., Whelan J., Claude L. (2015). Ewing Sarcoma: Current Management and Future Approaches Through Collaboration. J. Clin. Oncol..

[5] Isakoff M.S., Bielack S.S., Meltzer P.S., Gorlick R. (2015). Osteosarcoma: Current Treatment and a Collaborative Pathway to Success. J. Clin. Oncol..

[6] Cerny T., Castiglione M., Brunner K., Kupfer A., Martinelli G., Lind M. (1990). Ifosfamide by continuous infusion to prevent encephalopathy. Lancet.

[7] Palumbo R., Palmeri S., Antimi M., Gatti C., Raffo P., Villani G., Toma S. (1997). Phase II study of continuous-infusion high-dose ifosfamide in advanced and/or metastatic pretreated soft tissue sarcomas. Ann. Oncol..

[8] Gharote M.A. (2020). Is continuous infusion of high-dose ifosfamide, a safe option? Drug review. Int. J. Mol. Immuno Oncol..

[9] Perego G., Gregis F., Rossi L., Mazzoleni M., Nozza S., Nozza R., Gatti V.P. (2020). Continuous-infusion and outpatient setting: A chance for patients, a challenge for hospital pharmacists. J. Oncol. Pharm. Pract..

[10] Schoenike S.E., Dana W.J. (1990). Ifosfamide and mesna. Clin. Pharm..

[11] Sprangers B., Lapman S. (2020). The growing pains of ifosfamide. Clin. Kidney J..

[12] Lorigan P.C., Verweij J., Papai Z., Rodenhuis S., Le Cesne A., Leahy M.G., Radford J.A., Van Glabbeke M.M., Kirkpatrick A., Hogendoorn P.C.W. (2007). Phase III Trial of Two Investigational Schedules of Ifosfamide Compared With Standard-Dose Doxorubicin in Advanced or Metastatic Soft Tissue Sarcoma: A European Organisation for Research and Treatment of Cancer Soft Tissue and Bone Sarcoma Group Study. J. Clin. Oncol..

[13] Martin-Liberal J., Alam S., Constantinidou A., Fisher C., Khabra K., Messiou C., Olmos D., Mitchell S., Al-Muderis O., Miah A. (2013). Clinical Activity and Tolerability of a 14-Day Infusional Ifosfamide Schedule in Soft-Tissue Sarcoma. Sarcoma.

[14] Sanfilippo R., Bertulli R., Marrari A., Fumagalli E., Pilotti S., Morosi C., Messina A., Tos A.P.D., Gronchi A., Casali P.G. (2014). High-dose continuous-infusion ifosfamide in advanced well-differentiated/dedifferentiated liposarcoma. Clin. Sarcoma Res..

[15] Lee S.H., Chang M.H., Baek K.K., Han B., Lim T., Lee J., Park J.O. (2011). High-Dose Ifosfamide as Second- or Third-Line Chemotherapy in Refractory Bone and Soft Tissue Sarcoma Patients. Oncology.

[16] Singh A.S., Sankhala K.K., Mukherjee A., Narasimha V., Chmielowski B., Quon D.V., Chua V., Chawla S.P. (2014). 14-day continuous infusion ifosfamide in advanced refractory sarcomas. J. Clin. Oncol..

[17] Noujaim J., Constantinidou A., Messiou C., Thway K., Miah A., Benson C., Judson I., Jones R.L. (2015). Successful Ifosfamide Rechallenge in Soft-Tissue Sarcoma. Am. J. Clin. Oncol..

[18] Crago A.M., Singer S. (2011). Clinical and molecular approaches to well differentiated and dedifferentiated liposarcoma. Curr. Opin. Oncol..

[19] Spillane A.J., Fisher C., Thomas J.M. (1999). Myxoid liposarcoma—Frequency and the natural history of nonpulmonary soft tissue metastases. Ann. Surg. Oncol..

[20] Choi H., Charnsangavej C., Faria S.C., Macapinlac H.A., Burgess M.A., Patel S.R., Chen L.L., Podoloff D.A., Benjamin R.S. (2007). Correlation of Computed Tomography and Positron Emission Tomography in Patients With Metastatic Gastrointestinal Stromal Tumor Treated at a Single Institution With Imatinib Mesylate: Proposal of New Computed Tomography Response Criteria. J. Clin. Oncol..

[21] Stacchiotti S., Collini P., Messina A., Morosi C., Barisella M., Bertulli R., Piovesan C., Dileo P., Torri V., Gronchi A. (2009). High-Grade Soft-Tissue Sarcomas: Tumor Response Assessment—Pilot Study to Assess the Correlation between Radiologic and Pathologic Response by Using RECIST and Choi Criteria. Radiology.

[22] Wagner M.J., Livingston J.A., Patel S.R., Benjamin R. (2016). Chemotherapy for Bone Sarcoma in Adults. J. Oncol. Pract..

[23] Meazza C., Casanova M., Luksch R., Podda M., Favini F., Cefalo G., Massimino M., Ferrari A. (2010). Prolonged 14-day continuous infusion of high-dose ifosfamide with an external portable pump: Feasibility and efficacy in refractory pediatric sarcoma. Pediatr. Blood Cancer.

[24] Seddon B.M., McTiernan A.M., Michelagnoli M.P., Gabbie S., Daw S., Whelan J.S. High-dose ifosfamide in relapsed or progressive Ewing’s sarcoma. Proceedings of the Sarcoma Meeting Stuttgart.

[25] Ferrari S., Del Prever A.B., Palmerini E., Staals E., Berta M., Balladelli A., Picci P., Fagioli F., Bacci G., Vanel D. (2009). Response to high-dose ifosfamide in patients with advanced/recurrent Ewing sarcoma. Pediatr. Blood Cancer.

[26] McCabe M.G., Kirton L., Khan M., Fenwick N., Dirksen U., Gaspar N., Kanerva J., Kuehne T., Longhi A., Luksch R. (2020). Results of the second interim assessment of rEECur, an international randomized controlled trial of chemotherapy for the treatment of recurrent and primary refractory Ewing sarcoma (RR-ES). J. Clin. Oncol..

[27] Brain EC G., Mita A., Soulie P., Errihani H., Bessard A.H., Chaouche M., Alexandre J., Cvitkovic E., Jasmin C., Misset J.L. (1997). 6-day continuous infusion of high-dose ifosfamide with bone marrow growth factors in advanced refractory malignancies. J. Cancer Res. Clin. Oncol..

[28] Palmerini E., Setola E., Grignani G., D’Ambrosio L., Comandone A., Righi A., Longhi A., Cesari M., Paioli A., Hakim R. (2020). High Dose Ifosfamide in Relapsed and Unresectable High-Grade Osteosarcoma Patients: A Retrospective Series. Cells.

[29] Smith T.J., Bohlke K., Lyman G.H., Carson K.R., Crawford J., Cross S.J., Goldberg J.M., Khatcheressian J.L., Leighl N.B., Perkins C.L. (2015). Recommendations for the Use of WBC Growth Factors: American Society of Clinical Oncology Clinical Practice Guideline Update. J. Clin. Oncol..

[30] Authority H.R. Defining Research—National Research Ethics Service Guidance to Help You Decide If Your Project Requires Review by a Research Ethics Committee. April 2013. http://www.hra-decisiontools.org.uk.

